# Servant Leadership and Innovative Work Behavior in Chinese High-Tech Firms: A Moderated Mediation Model of Meaningful Work and Job Autonomy

**DOI:** 10.3389/fpsyg.2018.01767

**Published:** 2018-10-01

**Authors:** Wenjing Cai, Evgenia I. Lysova, Svetlana N. Khapova, Bart A. G. Bossink

**Affiliations:** ^1^School of Public Affairs, University of Sciences and Technology of China, Hefei, China; ^2^Department of Management and Organization, VU University Amsterdam, Amsterdam, Netherlands; ^3^Department of Science, Business and Innovation, VU University Amsterdam, Amsterdam, Netherlands

**Keywords:** servant leadership, meaningful work, job autonomy, innovative work behavior, high-tech firms

## Abstract

Scholars acknowledge the critical role of employee innovative work behavior (IWB) in facilitating organizational innovation in high-tech industries. However, the current knowledge is far from complete to paint a clear picture of how to evoke employee IWB in the Chinese high-tech industry. Many Chinese high-tech firms face a challenge moving from hierarchy-based leadership toward more employee-centered leadership styles, as the styles have different effects on employees’ IWB. This perspective may complement and sharpen the incomplete picture. Drawing on a dynamic componential model of creativity and innovation, this study proposes and tests a moderated mediation model that examines the hypothesized positive influence of servant leadership on employee IWB via meaningful work as well as the moderating role of job autonomy in this process. We collected data (*N* = 288) from three Chinese high-tech firms and found that employees’ perceptions of meaningful work mediate the relationship between servant leaders and IWB. We also found that this mediating relationship is conditional on the moderating role of job autonomy in the path from servant leadership to meaningful work. The results further show that the indirect effect of servant leadership on employee IWB via meaningful work exists only when job autonomy is high.

## Introduction

The high-tech industry is increasingly becoming a driving force of China’s innovation capability (e.g., Zhou et al., 2017). To maintain its technological advantage in the context of the global market, policy makers, and business leaders in China are seeking to develop practices that trigger employees to adopt innovative work behaviors (IWBs) and thereby integrate innovation more into the DNA of local high-tech firms (Huang et al., 2017). Consequently, academics have also been exploring how to promote employee IWB in the Chinese context (e.g., Tu and Lu, 2013; Leung et al., 2014; Ren and Zhang, 2015; Wang et al., 2015).

Although international research on employee IWB offers a wide range of explanations for factors that are likely to predict IWB (e.g., Agarwal et al., 2012), we posit herein that these findings do not fully explain employee IWB in high-tech Chinese industries for several reasons. First, Chinese firms often have commanding and authoritative management, which can impede innovative outcomes (e.g., Tsui, 2004; Tian and Sanchez, 2017); therefore, scholars in the leadership field suggest exploring servant leadership that prioritizes employees’ IWBs (e.g., Chen et al., 2015). Servant leadership refers to “developing employees to their fullest potential in the area of task effectiveness, community stewardship, self-motivation, and future leadership capabilities” (Liden et al., 2008, p. 162). Its emphasis on serving followers by caring and putting subordinates first is consistent with the changing requirements of current and future employee management in China (e.g., Tsui, 2004; Yu and Miller, 2005). When servant leaders demonstrate employee-centered leadership behaviors via a serving-others orientation, employees can develop the ability and desire to engage in innovative work (van Dierendonck, 2011; Liden et al., 2014; Panaccio et al., 2015). The necessity of exploring the role of servant leadership also lies in the theoretical arguments on the positive influence of more established and well-researched leadership styles (e.g., transformational leadership) in innovation management research (e.g., Banks et al., 2018). Compared with these traditional leadership approaches, servant leadership is changing the functioning of the hierarchical pyramid by concentrating on serving employees through caring and putting them first. Conceptually, servant leadership fits today’s innovation-oriented organizations that are devoted to assisting employees in exploiting their creative potential (e.g., Williams et al., 2017). In practice, because employees are often viewed as the main sources of organizational innovation, servant leadership can be vital to unlocking employees’ search for purpose and facilitating their growth toward innovative achievements (e.g., Yoshida et al., 2014).

Second, although scholars acknowledge that leaders may motivate employee IWB, the existing findings overlook the recent societal trend of employees pursuing meaningfulness in their work. Indeed, researchers have shown that experiencing meaningfulness in their work helps employees pursue their work goals and engage in fruitful activities (Steger and Dik, 2010; Martela and Pessi, 2018). Specific to our research context, because high-tech industries are often characterized as innovation-oriented, employees with meaningful work may be more motivated to address the challenges that come with innovation (Staw, 1990; Simonton, 1999). Specifically, research has indicated that people who experience meaningful work often feel intrinsically motivated (Amabile and Pratt, 2016), and as a result, positive reactions to addressing challenges and problems in an innovative way may be invoked (e.g., Amabile et al., 1996; Tu and Lu, 2013). For example, experiencing meaningful work may enable employees to feel that they are more likely to benefit the organization as they engage in innovation and thereby make an impact (Wrzesniewski and Dutton, 2001). In addition, they may be more likely to engage in innovative and creative behavior in the workplace (Grant and Berry, 2011). Moreover, researchers have examined leadership styles that evoke employee perceptions of meaningful work (e.g., Grant, 2012; Frieder et al., 2018) by illustrating that leader behaviors that highlight the value of an employee’s work direct his/her attention toward the positive and meaningful aspects of required tasks (Cohen-Meitar et al., 2009). Following this line of investigation, we propose that servant leadership enables employees to feel that their work is significant and meaningful because servant leaders prioritize employees’ needs by expressing concern and paying attention to personal development (Liden et al., 2014). Notwithstanding the benefits of meaningful work in innovative processes (Overell, 2008; Soane et al., 2013), to date, few empirical investigations have concentrated on such issues in this research area (Amabile and Pratt, 2016).

Finally, the situational leadership approach (Podsakoff and MacKenzie, 1997) suggests that the extent to which (servant) leaders effectively influence their followers may depend on certain conditions (Liden et al., 2014; Chen et al., 2015), especially the task characteristics (Kerr and Jermier, 1978; Mumford et al., 2002; Avolio et al., 2009). Task characteristics are manifold, as each employee performs his/her own set of tasks (Coelho and Augusto, 2010), and the tasks taken together determine the innovative activity in the organization (Shalley et al., 2000). From this perspective, to comprehensively understand employee IWB in high-tech sectors, it is crucial to explore whether and how job autonomy acts as a factor in determining the influence of leaders on employee innovative outcomes.

**FIGURE 1:**
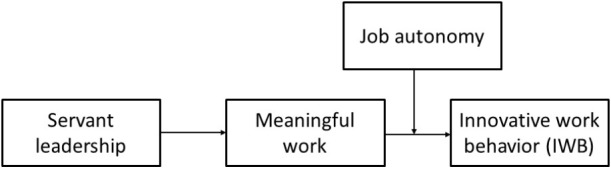
The hypothesized model.

We build on Amabile and Pratt’s (2016) dynamic componential model of creativity and innovation, which provides the theoretical insight that a desirable work environment (e.g., a positive leadership style) facilitates employee innovation via a motivating mechanism (e.g., meaningful work). In this study, we advance the existing theoretical and empirical research on employee IWB in the Chinese high-tech industry by examining the possibility that servant leadership fosters IWB by creating a more meaningful work experience among employees. Specifically, when servant leaders serve and create opportunities for employees’ development, the employees may tend to have a higher sense of purpose in their work (Greenleaf and Spears, 2002), and this may also increase the value employees attribute to innovation (Yuan and Woodman, 2010). Going further in considering a more autonomous context in which employees are provided more freedom and latitude, we propose a moderated mediation effect because autonomy may broaden employees’ choices (Ho and Nesbit, 2014) to fulfill their responsibility by completing their work in their own way (Langfred and Moye, 2004). Autonomy can stimulate employees to obtain more opportunities of being innovative from servant leadership (e.g., assistance and concern) in a meaningful way. The overall theoretical model visualizing this concept is presented in **Figure 1**.

Our study provides several contributions. First, examining the mediating role of meaningful work extends our understanding of how servant leadership can contribute to employee innovation from the perspective of positive individual attitudes (Mayer et al., 2008; Kool and van Dierendonck, 2012; Harwiki, 2013) instead of the well-researched perspective of identification constructs (van Dierendonck and Rook, 2010; Liden et al., 2014; Yoshida et al., 2014). In doing so, we answer calls to examine whether employees’ subjective experiences of meaningful work may transfer the benefits of contextual variables (e.g., leadership) to employee innovation (Cohen-Meitar et al., 2009; Amabile and Pratt, 2016). Furthermore, we enrich the theoretical arguments about when leadership styles generate a positive influence by examining job autonomy as a boundary condition in the indirect relation. We find empirical support for the contention that task characteristics strengthen leadership effects (Avolio et al., 2009; Wang and Cheng, 2010). In addition, we further illustrate that under the condition of high job autonomy, servant leadership may lead to employees’ perception of meaningful work and, in turn, their subsequent IWB. Finally, and of significant relevance for practice, we aim to enrich the concepts of servant leadership, meaningful work, and job autonomy in Chinese high-tech organizations. As one of the first initiatives to reconsider the traditional commanding and authoritative management paradigm in China (e.g., Tian and Sanchez, 2017; Tsui, 2004; Tsui et al., 2004; Warner, 1993), we specifically examine how servant leadership–unlike the well-researched traditional leadership styles that exert power from the top of the pyramid–drives subordinates toward innovation in a meaningful way. In doing so, our understanding of innovation-oriented leadership styles and task attributes is broadened, especially in Chinese high-tech sectors (e.g., Li et al., 1999; Sosik et al., 2005). Meanwhile, we aim to extend the current understanding of Chinese traditional settings by illustrating that an autonomous work structure, which is uncommon in most Chinese firms, has great potential to facilitate employee innovation (Wang and Cheng, 2010), especially in Chinese high-tech sectors.

### Servant Leadership and Meaningful Work

With the development of the focus on indigenous technological innovation in China, high-tech firms are increasingly requiring leaders to manage employee innovation effectively. However, traditional leadership styles are being questioned because they often lack a focus on the followers (Sendjaya and Sarros, 2002). The desire to investigate new follower-oriented leadership arises with the changes related to China’s current young generations, who increasingly appreciate leadership that facilitates them instead of commands them (e.g., designing innovative tasks and prioritizing individual needs). In terms of maintaining employees’ focus to enhance their motivation to innovate (van Dierendonck and Rook, 2010), servant leadership can nurture followers’ desirable outcomes (Barbuto and Wheeler, 2006; Mayer et al., 2008; Parris and Peachey, 2013). For example, Mayer et al. (2008) found that servant leadership is positively related to positive psychological states among employees (e.g., job satisfaction). Existing studies indicate that a servant leader can effectively fulfill subordinates’ needs by prioritizing their development (Chiniara and Bentein, 2016), which in turn can facilitate the creation of meaningful jobs (Owens and Hekman, 2012; Michaelson et al., 2014; van Dierendonck and Sousa, 2016). Nevertheless, this line of research primarily focuses on leadership behaviors’ influence on a single aspect of meaningful work in terms of job characteristics and overlooks the concept that work may also satisfy employees’ personal needs to connect with their surroundings and build relations with society and the community (Allan et al., 2014). Meaningful work represents “all that work means for individuals” and has a “significant and positive valence” (Steger et al., 2012, p. 323). Specifically, it is often argued that individuals’ perception of meaningful work refers to their subjective sense that their work has personal significance, contributes to a broader meaning of life (e.g., personal growth), and motivates them to positively influence others (Rosso et al., 2010).

This broader connotation of meaningful work in contemporary work environments (Pratt and Ashforth, 2003; Rosso et al., 2010) enables us to expect a positive relationship between servant leadership and meaningful work. To illustrate, when servant leaders put employees first, employees feel that they are valued as people (Liden et al., 2008). Moreover, when servant leaders’ behavior (e.g., providing assistance) cues employees that their work is worthwhile and significant, employees may develop a stronger sense that their work is meaningful and important. Similarly, servant leaders who set clear goals and build on employees’ strengths to achieve these goals can motivate employees to fulfill their responsibility with regard to goal realization (van Dierendonck, 2011). This responsibility can further develop individuals’ belief that their work is becoming more meaningful by aiming toward a desirable and shared organizational goal (Dik et al., 2009). In addition, servant leaders who prioritize subordinates’ personal growth (Bass, 2000), may increase the likelihood that their employees will have a meaningful work experience (Chiniara and Bentein, 2016). This line of reasoning suggests that servant leaders provide employees with assistance and support to enhance their skills and abilities (Liden et al., 2008), causing them to perceive their competence to be a powerful asset to develop and enjoy meaningful work (Gagné and Deci, 2005; van Dierendonck, 2011). Leaders’ servant behaviors can have a positive effect on subordinates’ behavior and can even make them, in turn, also start acting more as a servant employee (Greenleaf and Spears, 2002). Inspired by their servant leaders, employees may build a pro-social motivation that includes a strong desire to help others by displaying servant behavior themselves (Grant, 2007). Therefore, the employees may perceive that their work is meaningful work based on their perception of the impact they make at work (Steger and Dik, 2010). In short, servant leadership can leverage employees’ broader perception of meaningful work. Therefore, we propose:

*Hypothesis 1*: Servant leadership is positively related to employees’ perceptions of meaningful work.

### Meaningful Work and IWB

Following previous research that concludes that meaningful work is a predictor of employees’ desirable work outcomes (e.g., Steger et al., 2012), we propose a positive association between meaningful work and employee IWB, which is defined as a series of activities for the generation, promotion, and realization of ideas for new technologies, processes, techniques, or products (Janssen, 2000; Janssen and Van Yperen, 2004; Yuan and Woodman, 2010). Faced with the inevitable complexity of the technological innovation process, employees who believe that their work is meaningful may be internally motivated to manage the difficulties and challenges that come with this complexity (Staw, 1990; Simonton, 1999). The finding that people who experience their work as meaningful may personally invest in their work has been supported by a considerable number of studies (May et al., 2004). For example, a sense of meaningful work suggests that employees are intrinsically motivated to work (Amabile and Pratt, 2016; Steger et al., 2012) because they find purpose, value and significance in their tasks. Since they are intrinsically motivated, employees may tend to translate their motivation into a higher level of effort (e.g., generating, promoting and realizing their innovative activities) aimed at benefiting the organization in its (innovative) achievements (Amabile et al., 1996; Fuller et al., 2006; Yuan and Woodman, 2010; Tu and Lu, 2013).

The experience of meaningful work, which can include an individual’s perception of benefiting some greater good (Steger et al., 2012), may increase employees’ willingness to utilize their abilities and energies to make innovation achievements (Kashdan et al., 2004). By leading employees to find both significance and purpose in their work, and, thereby, to work for the sake of benefiting the organization’s (innovative) goals, leaders can encourage employees to exert more energy into their impact on their organization (Grant and Berry, 2011; Steger et al., 2012). As a result, these employees may be more likely to engage in innovative and creative activities for the benefit of the organization (e.g., coming up with, sharing, and applying novel ideas) (De Dreu and Nauta, 2009). In sum, we posit that employees’ perception of meaningful work will enhance their IWB. Therefore, we propose:

*Hypothesis 2*: Employees’ perceptions of meaningful work are positively related to their IWB.

### Meaningful Work as a Mediator

We further suggest that meaningful work can be a key mediator in explaining how servant leadership can contribute to employee IWB. According to the dynamic componential model of creativity and innovation (Amabile and Pratt, 2016), meaningful work acts as a mechanism that connects situational predictors and employee IWB. Prior research has found that innovation-stimulating leadership (e.g., transformational leadership) can facilitate subordinates’ desirable outcomes, including by making work more meaningful (Arnold et al., 2007; Walumbwa et al., 2013). As a leadership style that concentrates on generating meaning (van Dierendonck and Sousa, 2016), servant leadership may be positively related to employees’ IWB via their perception of meaningful work.

It has been argued that leaders’ behavior and attitude reflect the organization’s values and goals (Avolio et al., 2009). In organizations in which innovation is highly welcome, servant leaders can transfer their organizational innovation goals to subordinates (Liden et al., 2008), thus imbuing their work with meaning by connecting employees’ personal innovation goals with the broader organizational goals (Rosso et al., 2010). To realize employees’ personal goals, servant leadership can provide the services and resources to enable employees to engage in more innovative work. Moreover, servant leadership that emphasizes employees’ growth and development can help employees feel that they are living up to their full potential (Pratt and Ashforth, 2003). It can be argued that such employees are more likely to view their work as meaningful and to engage in innovative activities (Scott and Bruce, 1994). Accordingly, we propose that when a leader engages in servant behavior, employees are likely to perceive their work as meaningful, which motivates them to engage in innovative activities. Therefore, we propose as follows:

*Hypothesis 3*: Employees’ perceptions of meaningful work mediate the relationship between servant leadership and employee IWB.

### Job Autonomy as a Moderator

Job autonomy, defined as the extent to which individuals can decide on the methods, processes, and efforts necessary to accomplish their jobs/tasks (Hackman and Oldham, 1976), and is often presented as an important contextual factor in predicting employee creativity and innovation (Scott and Bruce, 1994; Liu et al., 2011). Many scholars have also found that job autonomy contributes to a high level of employee well-being (e.g., Thompson and Prottas, 2006); for example, when employees are performing autonomous tasks, they can freely pursue the interests and activities they value (Reis et al., 2000).

According to situational leadership theory, previous research suggests that job characteristics may determine the dysfunctional effects of leadership on employee well-being (e.g., Kalshoven and Boon, 2012). In line with this argument, we hypothesize that job autonomy moderates the association between servant leadership and meaningful work. Theoretically, job autonomy enables self-determination and meaning (Deci and Ryan, 2000; Niemiec et al., 2010), and when individuals have more opportunities to express themselves, they will perceive meaning in their work (e.g., May et al., 2004). Based on these arguments, it can be maintained that individuals who have a great deal of autonomy are provided the latitude to respond to their leaders’ behavior in the workplace (e.g., Wang and Cheng, 2010). Specifically, job autonomy helps employees feel more responsible for their work outcomes because they act as an agent with great latitude to manage their own work process (Hackman and Oldham, 1976). Thus, they are more likely to experience meaningfulness based on a sense of control (e.g., Rosso et al., 2010). In other words, when job autonomy is high, employees may have more opportunities to proactively receive and take advantage of the benefits of their servant leaders’ supervision to experience meaning in the workplace domain (Gagné and Deci, 2005; Den Hartog and Belschak, 2012; Ho and Nesbit, 2014). Thus, considerable autonomy among employees can strengthen the relationship between servant leadership and employees’ meaningful work.

In contrast, employees who have a low degree of autonomy in their jobs may gain a low level of psychological satisfaction. This can constrain individuals’ willingness to engage in desirable innovative behavior (e.g., Hackman and Oldham, 1976). Even though servant leaders often aim to create an atmosphere for employees to find meaning and purpose in their work, not all employees are likely to experience their activities as meaningful. In such situations in which employees feel less or no excitement, i.e., people who are not open to the experience of engaging in meaningful work (Coelho and Augusto, 2010), the servant leader’s effect on his/her employees may be very limited (Wang and Cheng, 2010; Volmer et al., 2012). In such a situation, employees may act more self-contained and may not be open to an interdependent working environment. They also may not have the ability to utilize the available resources provided by the servant leaders that can make their work more meaningful. Therefore, we propose:

*Hypothesis 4*: Job autonomy moderates the relationship between servant leadership and employees’ perceptions of meaningful work in such a way that this relationship is stronger in situations of high rather than low job autonomy.

Despite the impact of high or low levels of job autonomy on how employees respond to the effect of (different levels of) servant leadership with regard to their perception of engaging in meaningful work, levels of autonomy can be still critical in predicting levels of IWB. In this regard, ample research findings have indicated that successful innovative organizations (e.g., high-tech firms) are characterized as acting in a discretion-requirement context (Hambrick and Finkelstein, 1987) in which a wide latitude of choices can efficiently enable employees to try innovative solutions and develop innovative products (e.g., Scott and Bruce, 1994; De Jong and Den Hartog, 2010; Orth and Volmer, 2017). In other words, job autonomy may moderate not only the relationship between servant leadership and meaningful work but also the indirect relation between servant leadership and IWB through meaningful work. Specifically, when a high level of job autonomy broadens employees’ choices (Ho and Nesbit, 2014) and renders them more responsible for their work (Langfred and Moye, 2004), it can motivate greater IWB based on a meaningful response to the benefits of servant leadership. Therefore, the indirect effect of servant leadership on employee IWB through meaningful work is likely to be stronger in situations with a high level of job autonomy. However, when employees are constrained by a low level of job autonomy, they may have fewer opportunities to utilize the benefits of servant leadership to build their perception of meaningful work; consequently, the indirect effect of servant leadership on employee IWB may be weaker. Accordingly, we propose:

*Hypothesis 5*: Job autonomy moderates the indirect effect of servant leadership on IWB through meaningful work in such a way that the indirect effect is stronger when job autonomy is high.

## Materials and Methods

### Samples and Procedures

Data collection was conducted in three Chinese high-tech firms by using a questionnaire survey designed for this study. These firms are comparable with regard to several basic organizational characteristics. They are all IT-oriented organizations of a similar size and structure in southwest China. We first contacted the CEOs/HR officers of these companies to confirm that employee innovation was welcomed and to obtain permission for our investigation. Next, employees completed the questionnaires online after a brief introduction explaining that all information provided would be kept confidential, and the results would be sent to the researchers only. To avoid response bias, the names of the measures were not revealed, and the survey was anonymous. Informed consent was obtained from all participants to ensure that the researchers had the right to use the collected data. The original questionnaires were written in English; we used a back-translation process (Brislin, 1986) to create a Chinese version.

The sample included 288 participants, 54.5% of whom were male and 45.5% of whom were female. The average age of the employees was 30.5 years, and the majority had at least a bachelor’s degree (55.9%). Participants’ average number of years working in the relevant company was 3.3 years.

### Measures

Existing measures with established validity and reliability were used to operationalize all constructs in our study. We used a five-point Likert-type scale (from 1 = “strongly disagree” to 5 = “strongly agree”) to rate all items.

### Servant Leadership

Employees evaluated their managers’ servant leadership on a 7-item scale developed by Liden et al. (2014) (χ^2^ [11] = 16.98; TLI = 0.99; CFI = 0.99; RMSEA = 0.04). This scale measures the extent to which employees perceive their supervisor’s servant leadership style in organizations. A sample item is “my manager puts my best interests ahead of his/her own” (α = 0.86).

### Meaningful Work

Meaningful work was assessed using the Work and Meaning Inventory (WAMI) with ten items (Steger et al., 2012) (χ^2^ [24] = 33.25; TLI = 0.99; CFI = 0.98; RMSEA = 0.04). Employees rated the extent to which their work has a significant, worthwhile, and positive meaning (α = 0.89). Sample items are: “I have a good sense of what makes my job meaningful” (positive meaning); “I view my work as contributing to my personal growth” (meaning-making through work); and “The work I do serves a greater purpose” (greater good motivations). A higher-order factor of meaningful work was also found, suggesting that the total score can be used as an entire measurement.

### Job Autonomy

Job autonomy was assessed with two items by adopting the measure from the Job Diagnostic Survey (Hackman and Oldham, 1975). One reversed item was dropped because of a poor fit. Employees indicated the extent to which they have freedom, independence, and discretion to determine their jobs and tasks. A sample item is “I decide on my own how to go about doing the work” (α = 0.74).

### Innovative Work Behavior (IWB)

Six items from Scott and Bruce (1994) were used to evaluate employees’ IWB (χ^2^ [3] = 3.57; TLI = 0.99; CFI = 0.99; RMSEA = 0.02). We followed prior research and used self-reported ratings to ask employees to assess their IWB (e.g., Montani et al., 2014). A sample item is “I searched out new technologies, processes, techniques, and/or products” (α = 0.89).

### Control Variables

Following previous research (e.g., Scott and Bruce, 1994; Cuyper and Witte, 2006; Steger et al., 2013), we controlled for a number of demographic characteristics that may potentially influence the results in this study: age (in years), gender (1 = male; 2 = female), education (1 = high school, 5 = doctorate), and work tenure in the relevant organization (in years).

### Analytical Strategy

We tested our hypotheses using a PROCESS program developed by Preacher et al. (2007) in SPSS because it facilitates path analysis-based moderation and mediation analyses as well as their combination as a “conditional process model” by using ordinary least squares (OLS) regression. We used bootstrapping (10,000 samples) to analyze the extent to which servant leadership indirectly relates to employee IWB through meaningful work and whether this indirect effect is moderated by job autonomy. Specifically, to test the mediation effect, we used Model 4 in PROCESS, which generates direct and indirect effects in mediation. In this procedure, total effects, direct effects and indirect effects are estimated by means of OLS regression analyses. The effect of the independent variable (servant leadership) is displayed in the total effect; when controlling for the mediator variable (meaningful work), it is indicated in the direct effect. The indirect effect comprises the path from servant leadership to employee IWB through meaningful work. Providing accelerated confidence intervals through bootstrapping mitigates power problems and offers a more reliable estimation than the traditional Sobel test (Sobel, 1986) or the causal step method by Baron and Kenny (1986) for testing indirect effects. Next, to examine the moderated mediation effects, we first used Model 1 in the PROCESS program to examine whether the interactive effects (i.e., servant leadership × job autonomy) are significantly related to meaningful work. Next, we used Model 7 in the PROCESS program to obtain bias-corrected bootstrapped confidence intervals for the conditional indirect effect. We also bootstrapped with 10,000 iterations in order to generate bias-corrected confidence intervals for the significance tests of the conditional indirect effects (95% CIs) in the moderated mediation models (Hayes, 2013).

## Results

We conducted a confirmatory factor analysis (CFA) to assess the discriminant validity of measures in our study (Anderson and Gerbing, 1988). The results supports the finding that our hypothesized four-factor model demonstrated a better fit to the data (χ^2^ [231] = 389.14; TLI = 0.96; CFI = 0.96; RMSEA = 0.05) than all the alternative models (Hu and Bentler, 1999).

Since all the data were collected from employees at a single point in time, we applied two methods to identify the potential for common method bias (CMB). First, according to the explanatory factor analysis (Harman, 1976), the results showed that one factor accounted for 33.80%, which is below the accepted threshold of 40%. Second, we conducted the test of the one-factor measurement model (Podsakoff and Organ, 1986), which generated a poor fit to the data. Thus, CMB is not a serious problem in our study.

**Appendix Table A1** presents the descriptive statistics, correlations, and scale reliabilities for the research variables.

### Hypotheses Testing

**Appendix Table A2** presents the results of the hypotheses testing with regard to the mediation effects. To test H1, we examined the positive association between servant leadership and employees’ perception of meaningful work. The results show that after controlling for the effect of employees’ gender, age, education, and tenure, servant leadership was found to be significantly and positively related to employees’ perception of meaningful work (*β* = 0.37, *p* < 0.001). Thus, H1 was supported. Additionally, meaningful work was found to be positively related to employee IWB (*β* = 0.48, *p* < 0.001), which supports H2.

H3 suggests that meaningful work mediates the relationship between servant leadership and employee IWB. In **Appendix Table A2**, the results show that when servant leadership and meaningful work were simultaneously considered, the relationship between servant leadership and employee IWB was not significant (*β* = -0.04, *p* = 0.43). To further clarify the mediation effect, we used a bootstrap procedure with 10,000 samples to produce a confidence interval (CI) for the indirect effect. The results in **Appendix Table A3** reveal that the indirect effect through meaningful work was significant (*indirect effect* = 0.18, *p* < 0.01, 95% CI [0.12;0.26]). Therefore, H3 was fully supported.

**FIGURE 2:**
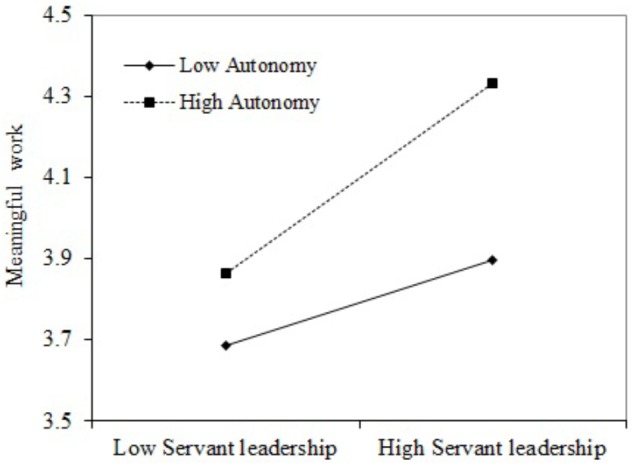
Moderating effects of autonomy on the servant leadership-meaningful work relationship.

H4 predicts that job autonomy moderates the relationship between servant leadership and meaningful work. As shown in **Appendix Table A4**, the interaction between servant leadership and job autonomy was positively related to meaningful work (*β* = 0.12, *p* < 0.05). As suggested by Aiken et al. (1991), we illustrated the pattern of the interaction effect. **Figure 2** depicts the plot of the moderation effect, showing that job autonomy significantly strengthens the relation between servant leadership and employees’ perception of meaningful work. We also conducted a simple slope test. Specifically, the relation between servant leadership and employees’ perception of meaningful work is stronger when job autonomy is high (*simple slope* = 0.35, *SE* = 0.05, *t* = 6.64, *p* < 0.001) than when job autonomy is low (*simple slope* = 0.16, *SE* = 0.07, *t* = 2.20, *p* < 0.05). Thus, H4 was supported.

H5 predicts that the indirect relation between servant leadership and employee IWB through meaningful work is conditional on the moderator variable of job autonomy for the path from servant leadership to meaningful work. Conducting a bootstrapping procedure with 10,000 samples, we estimated the conditional indirect effect of an independent variable (servant leadership) through a mediator (meaningful work) on a dependent variable (employee IWB) at both high and low levels of the moderator (job autonomy). The results of the bias-corrected confidence intervals in **Appendix Table A5** show that the indirect relation between servant leadership and employee IWB through meaningful work is significant only when job autonomy is higher (*indirect effect* = 0.17, 95% CI [0.11;0.26]), but not when job autonomy is lower (*indirect effect* = 0.08, 95% CI [-0.01;0.17]). The index of moderated mediation is 0.06 (SE = 0.03, 95% CI [0.001;0.118]). These results lend support for H5.

## Discussion

The present research extends the servant leadership-IWB association by establishing meaningful work as an intervening mechanism and autonomy as a boundary condition. As expected, we found that the indirect influence of servant leadership on employee IWB is mediated by employees’ perception of meaningful work and moderated by job autonomy. The findings illustrate that servant leadership has some influence on employee innovation by promoting meaningful work when job autonomy is high.

### Theoretical Implications

Our findings are consistent with prior research that acknowledges an indirect link between servant leadership and employee innovativeness (Neubert et al., 2008; Yoshida et al., 2014). We answered the call to explore the mechanisms between servant leadership and employee outcomes, especially innovation results (e.g., van Dierendonck and Rook, 2010; Liden et al., 2014; Yoshida et al., 2014). Extending existing research that suggests that an identity perspective transmits the impact of servant leadership on subordinates, our examination explores leadership’s effect on employees’ internal psychological process (Chiniara and Bentein, 2016) of perceiving work as meaningful and thereby to engage in innovation. The results show that when servant leaders nurture employees’ experience of meaningful work, employees feel motivated to be innovative; thus, we provide a new empirical contribution to the dynamic componential model of creativity and innovation (Amabile and Pratt, 2016). This approach broadens the scope of research on leaders as a source of meaningful work (e.g., Arnold et al., 2007; Walumbwa et al., 2013; Peng et al., 2016) by answering the call of Michaelson et al. (2014) to investigate the association between servant leadership styles and followers’ meaningful work (van Dierendonck et al., 2014; van Dierendonck and Sousa, 2016). In addition, we extend the prominent motivational perspective in creativity and innovation research (Liu et al., 2016). That is, meaningful work is a new type of motivational mediator that links contextual factors (e.g., leadership) with employee innovation results (Staw, 1990; Cohen-Meitar et al., 2009; Amabile and Pratt, 2016). Since previous research has suggested some concepts that constitute the mechanisms linking servant leadership with employee innovation outcomes, it would be productive for future research to explore other potential mechanisms (e.g., trust, engagement, and efficacy beliefs) (e.g., Sendjaya and Sarros, 2002; Walumbwa et al., 2010).

Job autonomy as revealed herein contributes to the servant leadership literature by suggesting that job autonomy significantly strengthens the relationship between servant leadership and meaningful work. More importantly, by answering calls for more research and extending past research on how context may enhance or inhibit the effects of servant leadership on employee innovation (e.g., Liden et al., 2014; Yoshida et al., 2014; Ling et al., 2016), this study indicates that job autonomy moderates the indirect relation between servant leadership and employee IWB through meaningful work, with slightly mixed implications. Regarding the integrated model, meaningful work mediates the relation between servant leadership and IWB only when employees have high job autonomy, not when they have low job autonomy. This finding suggests that the existence of a servant leadership style alone may be not enough for employees to experience meaningful work and IWB. Job autonomy is an important facilitator to boost the desirable outcomes of employees. That is, when highly autonomous jobs are assigned to employees, servant leadership may generate more benefits to enhance their meaningful work and thereby IWB. When employees have a low level of autonomy, regardless of whether servant leaders provide desirable behaviors that allow people to perceive their work as meaningful, they are unlikely to engage in innovation. In other words, high job autonomy is essential to determine whether servant leadership positively relates to meaningful work and IWB.

Our model, which is derived from Western theories, was specifically tested in the Chinese high-tech industry. Extending previous research, our study sheds new light on managing Chinese employees’ innovation by providing an ideal and specific setting for identifying and examining how servant leadership, meaningful work, and job autonomy together foster employee IWB. Specifically, since modern high-tech organizations are often less hierarchically structured, the role of leaders and leadership is changing from leading to serving employees, and employees’ tasks should be more autonomous to evoke their gratitude in innovation processes. To provide an updated and a more comprehensive understanding of employee innovation in China, research and practice can focus on an employee-centered leadership style,i.e., servant leadership (Walumbwa et al., 2010; Yoshida et al., 2014), since servant leaders can benefit employees’ growth in capabilities and help them develop a greater sense of meaningfulness (Yoshida et al., 2014). Moreover, granting greater job autonomy may enable individuals to engage in more self-determined activities aimed at exploring possibilities to generate and realize new ideas (Shalley et al., 2000; Wang and Cheng, 2010). All these findings seem appropriate and valuable for academics and managers to consider how to break down the traditional Chinese management philosophy (e.g., Tsui, 2004; Tian and Sanchez, 2017) and stimulate employees to behave in innovative ways in Chinese high-tech organizations, where indigenous innovation is increasingly valued and required.

### Practical Implications

Our research highlighted the significance of servant leadership and meaningful work in promoting employee IWB. Although most Chinese companies have a more hierarchical structure than Western countries (Redding, 1990), servant leadership has a positive influence in Chinese contexts, as in Western contexts (Han et al., 2010), with powerful effects on managing employees’ positive psychological states and innovativeness. Based on our findings, we discuss some managerial implications. First, the results suggest that leaders who serve subordinates motivate them to pursue meaningful work and innovative endeavors; therefore, organizations should select managers with servant leadership skills. Alternatively, supervisors can learn how to develop coaching and serving skills to benefit employees’ well-being, especially as it relates to their perception of meaningful work. In addition, organizations should adopt a servant leadership philosophy and establish servant leadership requirements for managers to develop leaders with a key “servant” orientation and mindset (e.g., emphasizing concerns for followers and prioritizing meaningfulness and innovation among employees). Second, given the importance of meaningful work, diverse human resource management practices should be implemented to help increase the meaningfulness of work (Grant, 2008). Specifically, by providing training programs relevant to employees’ work and by designing meaningful work for employees, an organization can effectively reinforce employees’ ability to find meaning in their jobs. Finally, considering the critical role of autonomy, a desirable social environment is required to strengthen the positive role of the IWB predictors. Specifically, managers should adopt an autonomy-oriented style, and organizations should also design autonomous work structures in the workplace that reinforce the advantages of servant leadership and its effect on meaningful work. Meanwhile, organizations should build an autonomous work environment to promote employees’ vitality in contributing to innovative outcomes.

### Limitations and Future Research Directions

The scope of our study is limited, but it does provide several insights for future research. First, our cross-sectional research design generates a problem of causality in our results. In the future, longitudinal designs should be conducted to provide evidence of reliable causalities. Second, we use self-reported rather than supervisor-rated assessments of employee IWB that may fail to assess it objectively. Even though scholars have found that employees can often provide a better judgment of their (innovative/creative) performance than supervisors because they (employees) have more information about their work activities (Janssen, 2000), we encourage future research to introduce more objective measures of IWB (e.g., measuring innovative work output and the work results of employees) and/or more balanced measures of IWB (e.g., including customer or supervisor assessments) (e.g., Stashevsky et al., 2006). In addition, given that IWB includes a series of stages (e.g., idea generation and idea implementation), the predictors may have different relations with separate aspects of the innovation process (Birdi et al., 2016). For example, in an empirical study by Birdi et al. (2016), social support had no significantly positive influence on idea implementation. Future research should examine whether the antecedents have the same effects on the different IWB stages.

Another aspect that relates to the limited scope of our research concerns the generalizability of our findings; this study examined a relatively small sample of IT companies. In this regard, it would be valuable to build on our findings using not only various other IT firms but also other types of industries/organizations to generate a larger dataset with more generalization value. Finally, since this research is situated in Chinese firms with a relatively strong focus on hierarchy and bounded individual autonomy, these findings may be less generalizable for firms in countries that are less hierarchical and that already provide higher levels of autonomy for their employees (e.g., Western European and United States firms). Future research is needed to examine which of our findings hold for these contexts as well.

## Ethics Statement

Data for this study was collected through an online survey. The survey questionnaire did not include any of the sensitive, personal privacy, ethical and moral topics. The survey questionnaire is available and can be provided upon a request. Moreover, data for this study was collected in 2016 and the ethics approval was not required at the time as per the university’s guidelines and national regulations.

Regarding the ethics of the data collection process, we complied with all ethical research rules necessary for a quantitative investigation. Specifically, to find participants for our study, we invited three high-tech firms from Sichuan Province in China to take part. Specifically, we first contacted the CEOs of these firms to explain the purpose of the study, and to invite their consent with regards to the firms’ participation. We explicitly asked them whether their organizations would like to join the study by doing the survey about servant leadership and employee IWBs. After receiving their consent, we asked CEOs to distribute the questionnaires to the employees in their companies. They appointed the human resource (HR) managers in the HR department of their organizations to send the online questionnaires to all employees. That is, the consent of the participants was obtained by virtue of survey completion after they were provided with sufficient information about the study. In the questionnaire, we clearly stated that “All data obtained through this survey will be kept confidentially and will only be used for research.” Moreover, the data was collected anonymously. This means that we do not have any identifying information regarding participants’ names.

Finally, the data resulting from this study is stored and protected according to the Data Management rules of the School of Business and Economics of the Vrije Universiteit Amsterdam.

## Author Contributions

All authors were responsible for the study conception and design. WC, EL, and SK organized the data collection. WC performed the data analysis. WC and EL were responsible for drafting the manuscript. SK and BB made critical revisions to the paper for important intellectual content.

## Conflict of Interest Statement

The authors declare that the research was conducted in the absence of any commercial or financial relationships that could be construed as a potential conflict of interest.

## Appendix

**Table A1:** Means, standard deviations, and correlations.

Variables	Mean	*SD*	1	2	3	4	5	6	7
1. Gender	1.45	0.50							
2. Age	30.54	6.37	-0.12^∗^						
3. Education	3.84	0.85	0.09	0.28^∗∗∗^					
4. Organizational tenure	3.33	3.13	-0.01	0.43^∗∗∗^	-0.14^∗^				
5. Servant leadership	3.61	0.66	0.03	-0.14^∗^	-0.03	-0.15^∗^			
6. Job autonomy	3.69	0.78	-0.10	-0.06	0.01	-0.04	0.37^∗∗^		
7. Meaningful work	3.82	0.56	-0.05	-0.07	-0.07	-0.15^∗^	0.45^∗∗^	0.37^∗∗^	
8. IWB	3.77	0.54	-0.21^∗∗^	0.03	-0.02	0.02	0.16^∗∗^	0.38^∗∗^	0.48^∗∗^

*N = 288; IWB = Innovative work behavior; ^∗^*p* < 0.05, and ^∗∗^*p* < 0.01*.

**Table A2:** Estimated coefficients for mediation model of meaningful work^a^.

	Outcome variable: meaningful work				Outcome variable: employee IWB			
	Coefficients	*SE*	*t*	95% CI	Coefficients	*SE*	*t*	95% CI
**Control variables**								
Gender	-0.05	0.06	-0.08	[-0.17,0.07]	-0.21^∗∗∗^	0.06	-0.70	[-0.32; -0.10]
Age	0.01	0.01	0.91	[-0.01,0.02]	-0.00	0.01	-0.31	[-0.01;0.01]
Education	-0.05	0.04	-1.43	[-0.13,0.02]	0.03	0.04	0.88	[-0.04;0.10]
Organizational tenure	-0.02^∗^	0.1	-1.97	[-0.04,0.00]	0.02	0.01	1.76	[-0.00;0.04]
**Independent variable**								
Servant leadership	0.37^∗∗∗^	0.05	8.12	[0.27,0.46]	-0.04	0.05	-0.79	[-0.13;0.06]
**Mediator**								
Meaningful work					0.48^∗∗∗^	0.06	8.89	[0.38;0.60]
Model summary^b^	R	R^2^	MSE	F	R	R^2^	MSE	F	
	0.46	0.22	0.25	15.47	0.53	0.28	0.22	18.00

*N = 288, ^∗^*p* < 0.05, ^∗∗^*p* < 0.01, and ^∗∗∗^*p* < 0.001. ^*a*^All statistics were performed using PROCESS (Model 4) in SPSS. ^*b*^All findings in the model(s) summary are significant (*p* < 0.001)*.

**Table A3:** Direct and indirect effects of servant leadership on employee IWB.

Direct effect of servant leadership on employee IWB		
**Effect**	***SE***	**t**	**95% CI**
-0.04	0.05	-0.79	[-0.13;0.06]

**Indirect effect servant leadership on employee IWB**			

**Effect**	**Boot *SE***	**Boot 95% CI**	
0.18	0.03	[0.12;0.26]	

*N = 288. All confidence intervals generated with bias corrected and accelerated bootstrapping (10,000 samples)*.

**Table A4:** Results of the moderation effects of job autonomy^a^.

	Outcome variable: meaningful work^a^				Outcome variable: employee IWB^b^			
	Coefficients	*SE*	*t*	95% CI	Coefficients	*SE*	*t*	95% CI
**Control variables**								
Gender	0.01	0.05	-0.22	[-0.13,0.10]	-0.21^∗∗∗^	0.06	-0.70	[-0.32, -0.10]
Age	0.01	0.01	1.03	[-0.01,0.02]	-0.00	0.01	-0.31	[-0.01,0.01]
Education	-0.06	0.04	-1.55	[-0.13,0.02]	0.03	0.04	0.88	[-0.04,0.10]
Organizational tenure	-0.02^∗^	0.01	-2.23	[-0.04,-0.00]	0.02	0.01	1.76	[-0.00,0.04]
**Independent variable**								
Servant leadership	-0.20	0.21	-0.97	[-0.61,0.21]	-0.04	0.05	-0.79	[-0.13,0.06]
**Mediator**								
Meaningful work					0.49^∗∗∗^	0.06	8.89	[0.38,0.60]
					-0.21^∗∗∗^	0.06	-0.70	[-0.32,-0.10]
**Interactive effect**					-0.00	0.01	-0.31	[-0.01,0.01]
Servant leadership × job autonomy	0.12^∗^	0.06	2.45	[0.02,0.22]				
Model summary ^c^	R	R^2^	MSE	F	R	R^2^	MSE	F
	0.53	0.28	0.23	15.41	0.53	0.28	0.22	18.00

*N* = 288, ^∗^*p* < 0.05, ^∗∗^*p* < 0.01, and ^∗∗∗^*p* < 0.001 ^*a*^All statistics were performed using PROCESS (Model 1) in SPSS. ^*b*^All statistics were performed using PROCESS (Model 7) in SPSS. ^*c*^All findings in the model(s) summary are significant (*p* < 0.001).

**Table A5:** Results of moderated mediation.

Moderator (job autonomy)	Indirect effect	*SE*	95% CI
Low levels of job autonomy (-1 *SD*)	0.08	0.05	[-0.01,0.17]
High levels of job autonomy (+1 *SD*)	0.17	0.04	[0.11,0.26]

*N = 288. All confidence intervals generated with bias corrected and accelerated bootstrapping (10,000 samples)*.

## References

[B1] Agarwal U. A. Datta S. Blake-Beard S. Bhargava S. (2012). Linking LMX, innovative work behaviour and turnover intentions The mediating role of work engagement. *Career Dev. Int.* 17 208–230. 10.1108/13620431211241063

[B2] Aiken L. S. West S. G. Reno R. R. (1991). *Multiple Regression: Testing and Interpreting Interactions.* Newbury Park, CA: Sage.

[B3] Allan B. A. Autin K. L. Duffy R. D. (2014). Examining social class and work meaning within the psychology of working framework. *J. Career Assess.* 22 543–561. 10.1177/1069072713514811

[B4] Amabile T. M. Conti R. Coon H. Lazenby J. Herron M. (1996). Assessing the work environment for creativity. *Acad. Manag. J.* 39 1154–1184. 10.5465/256995

[B5] Amabile T. M. Pratt M. G. (2016). The dynamic componential model of creativity and innovation in organizations: making progress, making meaning. *Res. Organ. Behav.* 36 157–183. 10.1016/j.riob.2016.10.001

[B6] Anderson J. C. Gerbing D. W. (1988). Structural equation modeling in practice: a review and recommended two-step approach. *Psychol. Bull.* 103 411–423. 10.1037/0033-2909.103.3.411

[B7] Arnold K. A. Turner N. Barling J. Kelloway E. K. Mckee M. C. (2007). Transformational leadership and psychological well-being: the mediating role of meaningful work. *J. Occup. Health Psychol.* 12 193–203. 10.1037/1076-8998.12.3.193 17638487

[B8] Avolio B. J. Walumbwa F. O. Weber T. J. (2009). Leadership: current theories, research, and future directions. *Annu. Rev. Psychol.* 60 421–449. 10.1146/annurev.psych.60.110707.163621 18651820

[B9] Banks G. C. Gooty J. Ross R. L. Williams C. E. Harrington N. T. (2018). Construct redundancy in leader behaviors: a review and agenda for the future. *Leadersh. Q.* 29 236–251. 10.1016/j.leaqua.2017.12.005

[B10] Barbuto J. E. Wheeler D. W. (2006). Scale development and construct clarification of servant leadership. *Group Organ. Manag.* 31 300–326. 10.1177/1059601106287091

[B11] Baron R. M. Kenny D. A. (1986). The moderator-mediator variable distinction in social psychological research: conceptual, strategic, and statistical considerations. *J. Pers. Soc. Psychol.* 51 1173–1182. 10.1037/0022-3514.51.6.1173 3806354

[B12] Bass B. M. (2000). The future of leadership in learning organizations. *J. Leadersh. Organ. Stud.* 7 18–40. 10.1177/107179190000700302

[B13] Birdi K. Leach D. Magadley W. (2016). The relationship of individual capabilities and environmental support with different facets of designers’ innovative behavior. *J. Prod. Innov. Manage.* 33 19–35. 10.1177/107179190000700302

[B14] Brislin R. W. (1986). “The wording and translation of research instruments,” in *Field Methods in Cross-Cultural Research*, eds Lonner W. J. Berry J. W. (Thousand Oaks, CA: Sage), 137–164.

[B15] Chen Z. Zhu J. Zhou M. (2015). How does a servant leader fuel the service fire? A multilevel model of servant leadership, individual self identity, group competition climate, and customer service performance. *J. Appl. Psychol.* 100 511–521. 10.1037/a0038036 25314366

[B16] Chiniara M. Bentein K. (2016). Linking servant leadership to individual performance: differentiating the mediating role of autonomy, competence and relatedness need satisfaction. *Leadersh. Q.* 27 124–141. 10.1016/j.leaqua.2015.08.004

[B17] Coelho F. Augusto M. (2010). Job characteristics and the creativity of frontline service employees. *J. Serv. Res.* 13 426–438. 10.1177/1094670510369379

[B18] Cohen-Meitar R. Carmeli A. Waldman D. A. (2009). Linking meaningfulness in the workplace to employee creativity: the intervening role of organizational identification and positive psychological experiences. *Creat. Res. J.* 21 361–375. 10.1080/10400410902969910

[B19] Cuyper N. Witte H. (2006). The impact of job insecurity and contract type on attitudes, well-being and behavioural reports: a psychological contract perspective. *J. Occup. Organ. Psychol.* 79 395–409. 10.1348/096317905X53660

[B20] Deci E. L. Ryan R. M. (2000). The “What” and “Why” of goal pursuits: human needs the self-determination of behavior. *Psychol. Inquiry* 11 227–268. 10.1207/S15327965PLI1104_01

[B21] De Dreu C. K. Nauta A. (2009). Self-interest and other-orientation in organizational behavior: implications for job performance, prosocial behavior, and personal initiative. *J. Appl. Psychol.* 94 913–926. 10.1037/a0014494 19594234

[B22] Den Hartog D. N. Belschak F. D. (2012). When does transformational leadership enhance employee proactive behavior? The role of autonomy and role breadth self-efficacy. *J. Appl. Psychol.* 97 194–202. 10.1037/a0024903 21842977

[B23] De Jong P. J. Den Hartog D. N. (2010). Measuring innovative work behaviour. *Creat. Innov. Manag.* 19 23–36. 10.1111/j.1467-8691.2010.00547.x

[B24] Dik B. J. Duffy R. D. Eldridge B. M. (2009). Calling and vocation in career counseling: recommendations for promoting meaningful work. *Prof. Psychol. Res. Pract.* 40 625–632. 10.1037/a0015547

[B25] Frieder R. E. Wang G. Oh I.-S. (2018). Linking job-relevant personality traits, transformational leadership, and job performance via perceived meaningfulness at work: a moderated mediation model. *J. Appl. Psychol.* 103 324–333. 10.1037/apl0000274 29016164

[B26] Fuller J. B. Marler L. E. Hester K. (2006). Promoting felt responsibility for constructive change and proactive behavior: exploring aspects of an elaborated model of work design. *J. Organ. Behav.* 27 1089–1120. 10.1002/job.408

[B27] Gagné M. Deci E. L. (2005). Self-determination theory and work motivation. *J. Organ. Behav.* 26 331–362. 10.1002/job.322

[B28] Grant A. M. (2008). Does intrinsic motivation fuel the prosocial fire? Motivational synergy in predicting persistence, performance, and productivity. *J. Appl. Psychol.* 93 48–58. 10.1037/0021-9010.93.1.48 18211134

[B29] Grant A. M. (2007). Relational job design and the motivation to make a prosocial difference. *Acad. Manag. Rev.* 32 393–417. 10.5465/amr.2007.24351328

[B30] Grant A. M. (2012). Leading with meaning: beneficiary contact, prosocial impact, and the performance effects of transformational leadership. *Acad. Manag. J.* 55 458–476. 10.5465/amj.2010.0588

[B31] Grant A. M. Berry J. W. (2011). The necessity of others is the mother of invention: intrinsic and prosocial motivations, perspective taking, and creativity. *Acad. Manag. J.* 54 73–96. 10.5465/AMJ.2009.43670890

[B32] Greenleaf R. K. Spears L. C. (2002). *Servant Leadership: A Journey into the Nature of Legitimate Power and Greatness.* Mahwah, NJ: Paulist Press.

[B33] Hackman J. R. Oldham G. R. (1975). Development of the job diagnostic survey. *J. Appl. Psychol.* 60 159–170. 10.1037/h0076546

[B34] Hackman J. R. Oldham G. R. (1976). Motivation through the design of work: test of a theory. *Organ. Behav. Hum. Perform.* 16 250–279. 10.1016/0030-5073(76)90016-7

[B35] Hambrick D. C. Finkelstein S. (1987). Managerial discretion: a bridge between polar views of organizational outcomes. *Res. Organ. Behav.* 9 369–406.

[B36] Han Y. Kakabadse N. K. Kakabadse A. (2010). Servant leadership in the people’s republic of China: a case study of the public sector. *J. Manag. Dev.* 29 265–281. 10.1108/02621711011025786

[B37] Harman H. H. (1976). *Modern Factor Analysis.* Chicago, IL: University of Chicago Press.

[B38] Harwiki W. (2013). The influence of servant leadership on organization culture, organizational commitment, organizational citizenship behavior and employees’ performance (study of outstanding cooperatives in East Java Province, Indonesia). *J. Econ. Behav. Stud.* 5 876–885.

[B39] Hayes A. F. (2013). *Introduction to Mediation, Moderation, and Conditional Process Analysis: A Regression-Based Approach.* New York, NY: Guilford Press.

[B40] Ho J. Nesbit P. L. (2014). Self-leadership in a Chinese context work outcomes and the moderating role of job autonomy. *Group Organ. Manag.* 39 389–415. 10.1177/1059601114539389

[B41] Hu L. T. Bentler P. M. (1999). Cutoff criteria for fit indexes in covariance structure analysis: conventional criteria versus new alternatives. *Struct. Equ. Modeling* 6 1–55. 10.1080/10705519909540118

[B42] Huang K. G.-L. Geng X. Wang H. (2017). Institutional regime shift in intellectual property rights and innovation strategies of firms in China. *Organ. Sci.* 28 355–377. 10.1287/orsc.2017.1117

[B43] Janssen O. (2000). Job demands, perceptions of effort-reward fairness and innovative work behaviour. *J. Occup. Organ. Psychol.* 73 287–302. 10.1348/096317900167038

[B44] Janssen O. Van Yperen N. W. (2004). Employees’ goal orientations, the quality of leader-member exchange, and the outcomes of job performance and job satisfaction. *Acad. Manag. J.* 47 368–384. 10.2307/20159587

[B45] Kalshoven K. Boon C. T. (2012). Ethical leadership, employee well-being, and helping. *J. Pers. Psychol.* 11 60–68. 10.1027/1866-5888/a000056

[B46] Kashdan T. B. Rose P. Fincham F. D. (2004). Curiosity and exploration: facilitating positive subjective experiences and personal growth opportunities. *J. Pers. Assess.* 82 291–305. 10.1207/s15327752jpa8203_05 15151805

[B47] Kerr S. Jermier J. M. (1978). Substitutes for leadership: their meaning and measurement. *Organ. Behav. Hum. Perform.* 22 375–403. 10.1016/0030-5073(78)90023-5

[B48] Kool M. van Dierendonck D. (2012). Servant leadership and commitment to change, the mediating role of justice and optimism. *J. Organ. Change Manag.* 25 422–433. 10.1108/09534811211228139

[B49] Langfred C. W. Moye N. A. (2004). Effects of task autonomy on performance: an extended model considering motivational, informational, and structural mechanisms. *J. Appl. Psychol.* 89 934–945. 10.1037/0021-9010.89.6.934 15584833

[B50] Leung K. Chen Z. J. Zhou F. Lim K. (2014). The role of relational orientation as measured by face and renqing in innovative behavior in China: an indigenous analysis. *Asia Pac. J. Manag.* 31 105–126. 10.1007/s10490-011-9277-1

[B51] Li J. Lam K. Qian G. (1999). High-tech industries and competitive advantage in emerging markets: a study of foreign telecommunications equipment firms in China. *J. High Technol. Manag. Res.* 10 295–312. 10.1016/S1047-8310(99)00013-9

[B52] Liden R. C. Wayne S. J. Liao C. Meuser J. D. (2014). Servant leadership and serving culture: influence on individual and unit performance. *Acad. Manag. J.* 57 1434–1452. 10.5465/amj.2013.0034

[B53] Liden R. C. Wayne S. J. Zhao H. Henderson D. (2008). Servant leadership: development of a multidimensional measure and multi-level assessment. *Leadersh. Q.* 19 161–177. 10.1016/j.leaqua.2008.01.006

[B54] Ling Q. Lin M. Wu X. (2016). The trickle-down effect of servant leadership on frontline employee service behaviors and performance: a multilevel study of Chinese hotels. *Tour. Manag.* 52 341–368. 10.1016/j.tourman.2015.07.008

[B55] Liu D. Chen X.-P. Yao X. (2011). From autonomy to creativity: a multilevel investigation of the mediating role of harmonious passion. *J. Appl. Psychol.* 96 294–309. 10.1037/a0021294 21058804

[B56] Liu D. Jiang K. Shalley C. E. Keem S. Zhou J. (2016). Motivational mechanisms of employee creativity: a meta-analytic examination and theoretical extension of the creativity literature. *Organ. Behav. Hum. Decis. Process.* 137 236–263. 10.1016/j.obhdp.2016.08.001

[B57] Martela F. Pessi A. B. (2018). Significant work is about self-realization and broader purpose: defining the key dimensions of meaningful work. *Front. Psychol.* 9:363. 10.3389/fpsyg.2018.00363 29632502 PMC5879150

[B58] May D. R. Gilson R. L. Harter L. M. (2004). The psychological conditions of meaningfulness, safety and availability and the engagement of the human spirit at work. *J. Occup. Organ. Psychol.* 77 11–37. 10.1348/096317904322915892

[B59] Mayer D. M. Bardes M. Piccolo R. F. (2008). Do servant-leaders help satisfy follower needs? An organizational justice perspective. *Eur. J. Work Organ. Psychol.* 17 180–197. 10.1080/13594320701743558

[B60] Michaelson C. Pratt M. G. Grant A. M. Dunn C. P. (2014). Meaningful work: connecting business ethics and organization studies. *J. Bus. Ethics* 121 77–90. 10.1007/s10551-013-1675-5

[B61] Montani F. Odoardi C. Battistelli A. (2014). Individual and contextual determinants of innovative work behaviour: proactive goal generation matters. *J. Occup. Organ. Psychol.* 87 645–670. 10.1111/joop.12066

[B62] Mumford M. D. Scott G. M. Gaddis B. Strange J. M. (2002). Leading creative people: orchestrating expertise and relationships. *Leadersh. Q.* 13 705–750. 10.1016/S1048-9843(02)00158-3

[B63] Neubert M. J. Kacmar K. M. Carlson D. S. Chonko L. B. Roberts J. A. (2008). Regulatory focus as a mediator of the influence of initiating structure and servant leadership on employee behavior. *J. Appl. Psychol.* 93 1220–1233. 10.1037/a0012695 19025244

[B64] Niemiec C. P. Ryan R. M. Deci E. L. (2010). Self-determination theory and the relation of autonomy to self-regulatory processes and personality development. *Handb. Personal. Self Regul.* 169–191. 10.1002/9781444318111.ch8

[B65] Overell S. (2008). *Inwardness: The Rise of Meaningful Work.* London: Work Foundation.

[B66] Orth M. Volmer J. (2017). Daily within-person effects of job autonomy and work engagement on innovative behaviour: the cross-level moderating role of creative self-efficacy. *Eur. J. Work Organ. Psychol.* 26 601–612. 10.1080/1359432X.2017.1332042

[B67] Owens B. P. Hekman D. R. (2012). Modeling how to grow: an inductive examination of humble leader behaviors, contingencies, and outcomes. *Acad. Manag. J.* 55 787–818. 10.5465/amj.2010.0441

[B68] Panaccio A. Henderson D. J. Liden R. C. Wayne S. J. Cao X. (2015). Toward an understanding of when and why servant leadership accounts for employee extra-role behaviors. *J. Bus. Psychol.* 30 657–675. 10.1007/s10869-014-9388-z

[B69] Parris D. L. Peachey J. W. (2013). A systematic literature review of servant leadership theory in organizational contexts. *J. Bus. Ethics* 113 377–393. 10.1007/s10551-012-1322-6

[B70] Peng A. C. Y. Lin H. E. Schaubroeck J. Mcdonough E. F. Hu B. M. Zhang A. G. (2016). CEO intellectual stimulation and employee work meaningfulness: the moderating role of organizational context. *Group Organ. Manag.* 41 203–231. 10.1177/1059601115592982

[B71] Podsakoff P. M. MacKenzie S. B. (1997). Kerr and Jermier’s substitutes for leadership model: background, empirical assessment, and suggestions for future research. *Leadersh. Q.* 8 117–132. 10.1016/S1048-9843(97)90012-6

[B72] Podsakoff P. M. Organ D. W. (1986). Self-reports in organizational research: problems and prospects. *J. Manag.* 12 531–544. 10.1177/014920638601200408 8452065

[B73] Pratt M. G. Ashforth B. E. (2003). “Fostering meaningfulness in working and at work,” in *Positive Organizational Scholarship: Foundations of a New Discipline*, eds Cameron K. S. Dutton J. E. Quinn R. E. (Oakland, CA: Berrett-Koehler), 309–327.

[B74] Preacher K. J. Rucker D. D. Hayes A. F. (2007). Addressing moderated mediation hypotheses: theory, methods, and prescriptions. *Multivariate Behav. Res.* 42 185–227. 10.1080/00273170701341316 26821081

[B75] Redding G. (1990). *The Spirit of Chinese Capitalism.* Berlin: Walter de Gruyter. 10.1515/9783110887709

[B76] Reis H. T. Sheldon K. M. Gable S. L. Roscoe J. Ryan R. M. (2000). Daily well-being: the role of autonomy, competence, and relatedness. *Personal. Soc. Psychol. Bull.* 26 419–435. 10.1177/0146167200266002

[B77] Ren F. Zhang J. (2015). Job stressors, organizational innovation climate, and employees’ innovative behavior. *Creat. Res. J.* 27 16–23. 10.1080/10400419.2015.992659

[B78] Rosso B. D. Dekas K. H. Wrzesniewski A. (2010). On the meaning of work: a theoretical integration and review. *Res. Organ. Behav.* 30 91–127. 10.1016/j.riob.2010.09.001

[B79] Scott S. G. Bruce R. A. (1994). Determinants of innovative behavior-A path model of individual innovation in the workplace. *Acad. Manag. J.* 37 580–607. 10.2307/256701

[B80] Sendjaya S. Sarros J. C. (2002). Servant leadership: its origin, development, and application in organizations. *J. Leadersh. Organ. Stud.* 9 57–64. 10.1177/107179190200900205

[B81] Shalley C. E. Gilson L. L. Blum T. C. (2000). Matching creativity requirements and the work environment: effects on satisfaction and intentions to leave. *Acad. Manag. J.* 43 215–223. 10.5465/1556378

[B82] Simonton D. K. (1999). *Origins of Genius: Darwinian Perspectives on Creativity.* Oxford: Oxford University Press.

[B83] Soane E. Shantz A. Alfes K. Truss C. Rees C. Gatenby M. (2013). The association of meaningfulness, well-being, and engagement with absenteeism: a moderated mediation model. *Hum. Resour. Manag.* 52 441–456. 10.1002/hrm.21534

[B84] Sobel M. E. (1986). Some new results on indirect effects and their standard errors in covariance structure models. *Sociol. Methodol.* 16 159–186. 10.2307/270922

[B85] Sosik J. J. Jung D. I. Berson Y. Dionne S. D. Jaussi K. S. (2005). Making all the right connections: the strategic leadership of top executives in high-tech organizations. *Organ. Dyn.* 34 47–61. 10.1016/j.orgdyn.2004.11.003

[B86] Stashevsky S. Burke R. Carmeli A. Meitar R. Weisberg J. (2006). Self-leadership skills and innovative behavior at work. *Int. J. Manpow.* 27 75–90. 10.1108/01437720610652853

[B87] Staw B. M. (1990). “An evolutionary approach to creativity and innovation,” in *Innovation and Creativity at Work: Psychological and Organizational Strategies*, eds West M. A. Farr J. L. (Oxford: John Wiley & Sons), 287–308.

[B88] Steger M. F. Dik B. J. (2010). “Work as meaning: Individual and organizational benefits of engaging in meaningful work,” in *Oxford Handbook of Positive Psychology and Work*, eds Linley P. A. Harrington S. Garcea N. (New York, NY: Oxford University Press), 131–142.

[B89] Steger M. F. Dik B. J. Duffy R. D. (2012). Measuring meaningful work: the work and meaning inventory (WAMI). *J. Career Assess.* 20 322–337. 10.1177/1069072711436160

[B90] Steger M. F. Littman-Ovadia H. Miller M. Menger L. Rothmann S. (2013). Engaging in work even when it is meaningless: positive affective disposition and meaningful work interact in relation to work engagement. *J. Career Assess.* 21 348–361. 10.1177/1069072712471517

[B91] Thompson C. A. Prottas D. J. (2006). Relationships among organizational family support, job autonomy, perceived control, and employee well-being. *J. Occup. Health Psychol.* 11 100–118. 10.1037/1076-8998.10.4.100 16551178

[B92] Tian Q. Sanchez J. I. (2017). Does paternalistic leadership promote innovative behavior? The interaction between authoritarianism and benevolence. *J. Appl. Soc. Psychol.* 47 235–246. 10.1111/jasp.12431

[B93] Tsui A. S. (2004). Contributing to global management knowledge: a case for high quality indigenous research. *Asia Pac. J. Manag.* 21 491–513. 10.1023/b:apjm.0000048715.35108.a7

[B94] Tsui A. S. Schoonhoven C. B. Meyer M. W. Lau C. M. Milkovich G. T. (2004). Organization and management in the midst of societal transformation: the People’s Republic of China. *Organ. Sci.* 15 133–144. 10.1287/orsc.104

[B95] Tu Y. Lu X. (2013). How ethical leadership influence employees’ innovative work behavior: a perspective of intrinsic motivation. *J. Bus. Ethics* 116 441–455. 10.1007/s10551-012-1455-7

[B96] van Dierendonck D. (2011). Servant leadership: a review and synthesis. *J. Manag.* 37 1228–1261. 10.1177/0149206310380462

[B97] van Dierendonck D. Rook L. (2010). *Enhancing Innovation and Creativity Through Servant Leadership,Servant Leadership.* Berlin: Springer,155–165.

[B98] van Dierendonck D. Sousa M. (2016). “Finding meaning in highly uncertain situations: servant leadership during change,” in *Leadership Lessons from Compelling Contexts*, eds Peus C. Braun S. Schyns B. (Bingley: Emerald Group Publishing Limited), 403–424.

[B99] van Dierendonck D. Stam D. Boersma P. De Windt N. Alkema J. (2014). Same difference? Exploring the differential mechanisms linking servant leadership and transformational leadership to follower outcomes. *Leadersh. Q.* 25 544–562. 10.1016/j.leaqua.2013.11.014

[B100] Volmer J. Spurk D. Niessen C. (2012). Leader–member exchange (LMX), job autonomy, and creative work involvement. *Leadersh. Q.* 23 456–465. 10.1016/j.leaqua.2011.10.005

[B101] Walumbwa F. O. Christensen A. L. Muchiri M. K. (2013). “Transformational leadership and meaningful work,” in *Purpose and Meaning in the Workplace*, eds Dik B. J. Byrne Z. S. Steger M. F. (Washington, DC: American Psychological Association), 10.1037/14183-010

[B102] Walumbwa F. O. Hartnell C. A. Oke A. (2010). Servant leadership, procedural justice climate, service climate, employee attitudes, and organizational citizenship behavior: a cross-level investigation. *J. Appl. Psychol.* 95 517–529. 10.1037/a0018867 20476830

[B103] Wang A. C. Cheng B. S. (2010). When does benevolent leadership lead to creativity? The moderating role of creative role identity and job autonomy. *J. Organ. Behav.* 31 106–121. 10.1002/job.634

[B104] Wang X. H. Fang Y. L. Qureshi I. Janssen O. (2015). Understanding employee innovative behavior: integrating the social network and leader-member exchange perspectives. *J. Organ. Behav.* 36 403–420. 10.1002/job.1994

[B105] Warner M. (1993). Human resource management ‘with Chinese characteristics’. *Int. J. Hum. Res. Manag.* 4 45–65. 10.1080/09585199300000004

[B106] Williams Jr Brandon R. S. Hayek M. Haden S. P. Atinc G. (2017). Servant leadership and followership creativity: the influence of workplace spirituality and political skill. *Leadersh. Organ. Dev. J.* 38 178–193. 10.1108/LODJ-02-2015-0019

[B107] Wrzesniewski A. Dutton J. E. (2001). Crafting a job: revisioning employees as active crafters of their work. *Acad. Manag. Rev.* 26 179–201. 10.5465/amr.2001.4378011

[B108] Yoshida D. T. Sendjaya S. Hirst G. Cooper B. (2014). Does servant leadership foster creativity and innovation? A multi-level mediation study of identification and prototypicality. *J. Bus. Res.* 67 1395–1404. 10.1016/j.jbusres.2013.08.013

[B109] Yu H.-C. Miller P. (2005). Leadership style: the x generation and baby boomers compared in different cultural contexts. *Leadersh. Organ. Dev. J.* 26 35–50. 10.1108/01437730510575570

[B110] Yuan F. R. Woodman R. W. (2010). Innovative behavior in the workplace: the role of performance and image outcome expectations. *Acad. Manag. J.* 53 323–342. 10.5465/amj.2010.49388995

[B111] Zhou K. Z. Gao G. Y. Zhao H. (2017). State ownership and firm innovation in China: an integrated view of institutional and efficiency logics. *Adm. Sci. Q.* 62 375–404. 10.1177/0001839216674457

